# Dental fear and anxiety and its sociodemographic associations among public and private schoolchildren aged 8 to 12 years in Depok, Indonesia: a cross-sectional study

**DOI:** 10.1186/s12903-026-08481-6

**Published:** 2026-05-11

**Authors:** Wisnu Fadila, Mugia Bayu Rahardja, Anne Agustina Suwargiani, Yanu Endar Prasetyo, Eriska Riyanti

**Affiliations:** 1 Doctoral Programme, Faculty of Dentistry, Universitas Padjadjaran, Bandung, Indonesia; 2 Research Centre for Population, National Research and Innovation Agency (BRIN), Jakarta Selatan, Indonesia; 3 Dental Public Health and Preventive Department, Faculty of Dentistry, Universitas Padjadjaran, Bandung, Indonesia; 4 Pediatric Dentistry Department, Faculty of Dentistry, Universitas Padjadjaran, Bandung, Indonesia

**Keywords:** Dental fear, Dental anxiety, Children, Sociodemographic factors, Indonesia

## Abstract

**Background:**

Dental fear and anxiety (DFA) affect dental visits and oral health outcomes in children. Sociodemographic factors linked to DFA are understudied in Indonesia.

**Objectives:**

This study examined the prevalence, triggers, and sociodemographic factors associated with DFA among schoolchildren aged 8–12 years in Depok City, Indonesia. The goal is to inform targeted interventions in school-based settings.

**Methods:**

A total of 460 schoolchildren aged 8–12 years were recruited from five elementary schools in Depok City by using randomised multistage cluster sampling. The participant’s parents also participated in the study. The questionnaires were adapted from the Indonesian Modified Dental Anxiety Scale with Facial Image Scale pictorial response aids (MDAS + FIS), Riskesdas, and Susenas and were administered by trained enumerators. The DFA scores were analysed as continuous and categorical data: no DFA (score 5), mild (6–11), and high (> 11). The covariates included age, sex, birth order, school grade, and school type. The statistical methods employed included descriptive statistics, linear regression, ordered logit regression, and generalised ordered logit regression. Sensitivity analyses for alternatives outcomes were conducted.

**Results:**

The mean DFA score was 11.1 (SD: 3.46), median 11 (IQR 9–13). In total, 53.2% of children had mild DFA, and 43.6% had high DFA. Injections (mean 3.13) and tooth drilling (mean 2.79) were the most common triggers. Older age (11–12 vs. 8–10 years), attending private schools, and higher birth order were consistently associated with lower DFA. Sex had weaker associations. Multivariate analysis confirmed these associations, with private schools and older age being the strongest predictors of reduced DFA. Sensitivity analyses affirmed robustness.

**Conclusions:**

There is a high prevalence of mild to high DFA. Early DFA strategies are needed to improve dental attendance and cooperation among Depok City schoolchildren. Lower DFA is associated with older age, later birth order, higher grades, and private schools. The major triggers were dental injections and drilling. These findings provide locally relevant evidence to tailor long-term oral health strategies.

**Supplementary Information:**

The online version contains supplementary material available at 10.1186/s12903-026-08481-6.

## Background

Dental fear and anxiety (DFA) refer to feelings of dread or apprehension related to dental treatment and are common barriers to optimal oral health care [1]. These experiences can lead to avoidance of dental visits, particularly during the mixed dentition period, when professional care is needed to maintain oral health and prevent future problems [2–6].

Rapid economic advancement has changed sociodemographic profiles in developing countries such as Indonesia, leading to increased prevalence of health-related problems, including dental caries [7]. A national survey substantiates this trend. It shows that more than half of children aged 5–14 years suffer from oral health problems, yet fewer than 15% receive professional dental care [8]. This low utilisation rate reflects not only structural barriers, such as limited access to dental services [9, 10], but also psychological barriers, such as DFA, which diminish cooperation and discourage attendance [11–13]. Furthermore, DFA is associated with children’s caries experience and oral health-related quality of life, underscoring its importance for physical and psychological well-being [13–16].

Evidence from international studies indicates that DFA in children arises from a combination of sociodemographic, experiential, and cognitive factors. DFA levels are associated with age, socioeconomic status, family characteristics, previous dental experiences, and specific dental procedures [5, 6, 17–27]. However, data on sociodemographic determinants among Indonesian schoolchildren, particularly in community and school-based settings, remain scarce. Previous studies have focused on non-schoolchildren [28–31]. To the best of our knowledge, no study has systematically examined the association of age, sex, birth order, school grade, and school type with DFA among Indonesian elementary schoolchildren.

In rapidly urbanising settings such as Depok City, which transformed from the outskirts of Jakarta to a major urban hub over three decades, school enrolment in public versus private schools often reflects differences in family sociodemographic backgrounds, parental involvement, and access to health information. These factors indicate that school type is a contextual factor influencing DFA. Understanding how age, sex, birth order, school grade, and school type relate to DFA in this population is important for developing targeted paediatric dental care strategies.

This study represents a focused component of a broader research project investigating DFA and its intervention among schoolchildren in Depok, Indonesia. The multiple determinants of DFA are investigated in this larger study. The current study specifically reports baseline findings on the prevalence of DFA, its triggers, and its association with selected sociodemographic attributes among children aged 8–12 years attending public and private elementary schools in Depok City, Indonesia. The results provide an initial understanding of sociodemographic factors influencing DFA and may guide future preventive strategies and behavioural interventions in paediatric dental care in the Indonesian context.

## Methods

### Study design and setting

This cross-sectional study was conducted between October and December 2024 in Depok City, Indonesia, as part of a larger project investigating determinants of DFA in children. Data were collected at a single time point across multiple schools. The aim was to assess DFA, its triggers, and sociodemographic associations. Public and private elementary schools participated to ensure diversity. Depok City was selected for its transition from an outskirt to an urban city, which offers diverse sociodemographic backgrounds and accessible school networks, improving representativeness. The child was the primary unit of analysis. Parental questionnaires provided additional sociodemographic context.

### Participants and sampling

The study included parent-child dyads with children aged 8–12 years attending public and private elementary schools in Depok City. The sample size was determined using the World Health Organisation guidelines for cross-sectional studies [32–34]. We assumed a 15% estimated prevalence (*p*) of DFA, based on a previous study [35], a 95% confidence level (*z* = 1.96), and a 5% precision (*d*). The sample size (*n*) was calculated using the formula *n* = *z*^2^*p*(1-*p*)/*d*^2^. The minimum estimated sample of 196 dyads was multiplied by a design effect of 2 and an expected data loss of 10%. This resulted in a total target of 432 dyads.

A multistage cluster random sampling design with simple random sampling was used at each stage (Table 1) [36]. Two subdistricts were selected from all 11 in Depok City by simple random sampling. The list of 42 eligible elementary schools (Limo: 9 public, 16 private; Cinere: 7 public, 10 private) was obtained from the Indonesian Ministry of Education’s *Dapodikdasmen* (*Data Pokok Pendidikan Dasar dan Menengah*/Primary and Secondary Education Core Data) national database. The database reports the total number of classes and pupil counts per school.

**Table 1:** Strobe flow: Multistage cluster sampling in elementary schools in Depok City

Stage	Sampling Unit	Eligible	Selected	Method/Notes
1	Subdistricts	11	2	Simple random sampling from all 11; Limo: 25 elementary schools (9 public, 16 private); Cinere: 17 elementary schools (7 public, 10 private) per 2023 *Dapodikdasmen*
2	Schools	42 (Limo: 25, Cinere: 17)	5 (3 public, 2 private)	Simple random sampling within the two selected subdistricts; schools with fewer than 12 classes and 20 pupils each were excluded and replaced through repeat random draws to maintain an adequate sample size and probability sampling
3	Classes (PSUs)	66 (grade 3–6)	27	Simple random sampling within the five selected schools.

Five schools were then selected using simple random sampling. Those that did not meet the minimum size criteria (≥12 classes with ≥20 pupils each) were excluded and replaced via repeat random draws to preserve probability sampling, yielding three public and two private schools. The exact number of eligible classes (grades 3–6) in each selected school was verified from the school’s official record. There were 66 eligible classes across these schools, of which 27 were selected via simple random sampling and served as primary sampling units (PSUs) for representativeness across public/private schools and subdistricts.

Inclusion criteria were as follows: all children aged 8–12 years who resided in Depok City with parent(s), did not currently undergo orthodontic treatment (to minimise measurement bias from recent dental exposure and altered dental perceptions), and did not live in institutional settings. This inclusive approach reflects the general elementary school population, including children with typical health conditions and learning difficulties. Dyads were excluded if either the parent or child questionnaire was incomplete.

Of the 804 parent-child dyads invited through letters, 496 (61.7%) returned completed consent forms. After screening for verification of residency, child age 8–12 years, and completeness of parent/child questionnaires, 36 were excluded. Six dyads residing outside Depok City (four from Krukut 1, two from Arrahman), one dyad not meeting the children’s age criterion (Gandul 1), 25 dyads due to unavailability of parents for the interview (22 from Gandul 1, one each from Cinere 1, Darul Ulum, and Arrahman), and four dyads with incomplete child responses (all from Darul Ulum). The final analysis included 460 dyads (Table 2). The overall response rate (57.2%) was calculated as the number of dyads that consented and were analysed (460) divided by the number of invited dyads (804), while the participation rate (92.7%) was calculated as the number of analysed dyads (460) divided by the number of consented dyads (496). The response rate was higher than that of typical independent surveys [36], which typically achieve lower response rates than large-scale government surveys.

**Table 2:** Participant flow summary

Stage	Description	Number (*n*)	Percentage (%)
Screening phase			
Initial invitation	Parent-child dyads invited from schools	804	100
Consented	Returned completed consent forms	496	61.7
	- Krukut 1 ES 0.7 (95/128)		
	- Cinere 1 ES 0.6 (92/160)		
	- Darul Ulum ES 0.7 (142/203)		
	- Arrahman ES 0.4 (44/115)		
	- Gandul 1 ES 0.6 (123/198)		
Exclusion phase			
Ineligible (residing area)	Did not meet residency criteria	6	0.8
Ineligible (age)	Outside 8–12 years age range	1	0.1
Parent unavailable	Unable to complete parent interview	25	3.1
Incomplete child response	Incomplete child questionnaire	4	0.5
Total excluded		36	4.4
Final analysis	Parent-child dyads in final sample	460	57.2

*ES* Elementary school

### Variables

The dependent variable was the DFA level, categorised using a median cut-off among children with DFA (total score > 5). Levels were “no DFA” (score = 5, all items “not anxious”), “mild DFA” (6–11), and “high DFA” (> 11). The independent variables were age group (8–10 vs. 11–12 years), sex (boys vs. girls), school grade (3, 4, 5, and 6), birth order (1, 2, ≥3), and school type (public vs. private). The dependent variable was derived from the child questionnaire, whereas the independent variables were derived from the child and parent questionnaires. Age was categorically analysed due to pronounced differences in mixed dentition stages and cognitive development between the ages of 8–10 and 11–12 years. School type served as a contextual proxy for socioeconomic context, with family-level variables (e.g., household expenditure, parental education) potentially confounding its association with DFA (Supplementary Appendix A). Family-level sociodemographic variables (e.g., household expenditure, parents’ education, parents’ age, and household compositions) were measured but excluded from the primary analyses. Sensitivity analyses included tertile-based DFA categorisation (low, mild, and high) derived from the total DFA score distribution and monthly household expenditure adjustment.

### Data source and measurement

Data were collected via enumerator-administered questionnaires to children and parents, adapted from Indonesia’s *Riskesdas* (*Riset Kesehatan Dasar*/The Indonesian Basic Health Research) and *Susenas* (*Survei Sosial Ekonomi Nasional*/The National Socioeconomic Survey) instruments [8, 37]. The *Riskesdas* is a nationally representative cross-sectional survey conducted by the Ministry of Health to monitor population health and service utilisation. The *Susenas* is a recurring household survey conducted by Statistics Indonesia that captures socioeconomic characteristics, welfare, and conditions at national and subnational levels. Questionnaire items were adapted from both surveys to be consistent with standard Indonesian sociodemographic and health-related variables.

The child questionnaire additionally incorporated the validated Indonesian Modified Dental Anxiety Scale (MDAS), comprising five dental scenario items (anticipating a dental visit, waiting in the clinic, undergoing tooth drilling, having teeth scaled or polished, and receiving a local anaesthetic injection) with corresponding response (ranging from 1, “not anxious”, to 5, “extremely anxious”), combined with the Facial Image Scale (FIS) for pictorial response options to form the MDAS + FIS instrument [38, 39]. This MDAS + FIS format enabled children’s responses via smiley-face visual aids during interviews (ranging from 1, “biggest smile/not anxious”, to 5, “extreme frown/extremely anxious”), a method that has been successfully used with children [26]. Items were adapted from the established Indonesian MDAS and were slightly modified for children’s comprehension during enumerator-administered interviews while preserving the original meaning [40]. The total DFA score is the sum of five items and ranges from 5 to 25. The complete MDAS + FIS block (Block IV of the child questionnaire) in both Bahasa Indonesia and English is provided in Supplementary Appendix B. Expert panel review confirmed the instrument’s contextual appropriateness before pilot testing.

### Instrument validity and reliability

Instrument validity and reliability were verified before fieldwork to minimise instrument-related bias. Procedures included expert review by three community dentistry experts for content validity (relevance and appropriateness), yielding a Content Validity Index (I-CVI) of 1.00 for all items in both child and parent questionnaires. A pilot study was conducted among fourth-grade children (*n* = 39) and parents (*n* = 32) from one unselected elementary school in Depok City. The test-retest interval for the child questionnaire was approximately 4 h on the same day, while for the parent questionnaire it was 6–8 h. Reliability was evaluated using Cohen’s kappa (*k*) for categorical items and polychoric correlations (*ρ*) for ordinal items such as Likert-type scales. The child questionnaire showed moderate to perfect agreement (*k* ≈ 0.60–1; all *p* < 0.001), the parent questionnaire showed predominantly perfect agreement (*k* ≈ 0.79–1; all *p* < 0.001), and the children’s DFA-related Likert items (*n* = 31) demonstrated strong to perfect latent consistency (*ρ* ≈ 0.829–1) with good model fit (Pearson G^2^ *p* ≥ 0.389). DFA scale internal reliability at Time 1 showed ordinal alpha = 0.77 and Cronbach’s alpha = 0.70. These parameters confirmed both temporal and construct reliability of the adapted instruments (see Supplementary Appendix C).

### Data collection procedures

Before data collection, official approval and research permits were obtained from the Depok City Education Office and the Depok City National Unity and Politics Agency. After the letters of permission were issued, meetings were conducted with school administrators and parent committees to explain study procedures. Research information sheets and informed consent forms were distributed to all parents.

Data collection was supervised by six trained supervisors and conducted by 16 trained enumerators (public health university students) who received three days of standardised training covering study protocols, questionnaire administration, and interviewer techniques, using uniform protocols to minimise interviewer bias. Children were interviewed in person by enumerators at schools for a more familiar environment, while parents were interviewed by telephone. To reduce response bias, enumerators were blinded to study hypotheses. Supervisors closely monitored field activities to ensure adherence to protocol, including random cognitive probing with multiple children at each school to verify their understanding of the questions, and random checks of interviews with participating parents to assess quality. Anonymity and confidentiality were emphasised throughout data collection to ensure genuine responses. Data quality logs and double-entry verification were implemented to reduce transcription errors.

### Statistical analysis

Data were entered into Stata 15.1 (StataCorp, College Station, TX, USA). The complex survey design was incorporated via the svyset command [41, 42], with all analyses using survey weights. The primary sampling units (PSUs) were classes, with schools and subdistricts as higher-level clusters.

Stratification was based on school type (public vs. private), and probability proportional to size (PPS) weighting was applied at the subdistrict, school, and class levels, using pupil count as the size measure. The finite population correction (FPC) was applied at the subdistrict stage. Base design weights were calibrated against Depok City’s school-age population estimates from the *Dapodikdasmen*, then adjusted by stratifying by subdistrict, grade, and sex. Due to the complete exclusion of nonrespondents, cell-based nonresponse weighting was not applied (see Supplementary Appendix D).

Descriptive statistics summarised sample characteristics and individual MDAS item scores. Descriptive associations between total DFA score and sociodemographic variables were explored using survey-weighted crosstabulations (*svy: tabulate*), linear regression, ordered logit, and generalised ordered logit. Linear regression of continuous DFA total scores served as the primary analysis, with model assumptions tested for homoscedasticity (residuals vs. fitted values) and normality (Q-Q plot and residuals histogram). Secondary categorical analyses used ordered logit (*svy: ologit*) and generalised ordered logit (*svy: gologit2*) models due to proportional odds assumption violations [43]. Sensitivity analyses adjusted models for monthly household expenditure and (for *gologit2*) tertile-based cut-offs. Missing data were handled via complete-case analysis (listwise deletion).

### Ethical considerations

Ethical approval was obtained from the Research Ethics Committee of Universitas Padjadjaran (Approval No. 1087/UN6.KEP/EC/2024), which complied with the Declaration of Helsinki. Only dyads with complete documentation were included as candidates in the study. Written informed consent was obtained from parents for both their own and their child’s participation, along with child assent. Participation was voluntary, and confidentiality was maintained by removing personal information before analysis. Incentives were small tokens to acknowledge participants’ time without coercion, and were distributed after data collection. Data were stored in password-protected files accessible only to the principal investigator.

## Results

After screening/exclusion and complete-case analysis (listwise deletion) of missing data, 460 children of 496 parent-child dyads (92.7%) were included in the final analysis. Of 129 variables (original, merged, and derived), 78 (60.5%) had missing data (mostly from 25 parent-questionnaire observations and four child-dental-examination observations each). For each affected variable, more than 94% of observations remained complete. Missing cases concentrated in 29 dyads, representing 2.6% of all dataset cells. No missing values remained in the total DFA score or any model variables. Survey weights were calibrated to Depok City grades 3–6 pupils (~ 2/3 of the grades 1–6 population).

Participant characteristics are shown in Table 3. Children were aged 8 to 12 years, with most (61.9%, *n* = 267) aged 8–10 years and nearly equal sex distribution (52.5% boys, *n* = 239). Most children attend public schools (62.9%, *n* = 282), were first-born (51.2%, *n* = 232), and were in grade 4 (39.2%, *n* = 178).

**Table 3:** Sociodemographic characteristics of study participants

Characteristics		*n*	% weighted
Age	8–10	267	61.9
	11–12	193	38.1
Sex	Boys	239	52.5
	Girls	221	47.5
Grade levels	3	68	17.2
	4	178	39.2
	5	105	22.2
	6	109	21.4
Birth order	1	232	51.2
	2	168	36.3
	3 or more	55	12.5
School type	Public	282	62.9
	Private	178	37.2
DFA levels	No DFA	16	3.1
	Mild DFA	246	53.2
	High DFA	198	43.6

*DFA* Dental fear and anxiety

Descriptive distribution of DFA scores showed a median of 11, with an interquartile range of 9–13 and a right-skewed distribution (Supplementary Appendix E). Overall, 3.1% of children reported no DFA (scores = 5), 53.2% had mild DFA (scores = 6–11), and 43.6% high DFA (scores > 11), based on the median cut-off applied among those with any DFA (score > 5). The mean total score was 11.07±3.46 (score range: 5–25), indicating a mild overall level of DFA among the participants (Table 4). The highest mean score among five items was associated with the local anaesthetic injection scenario (mean: 3.13, score range: 1–5), followed by tooth drilling (mean: 2.79, score range: 1–5). The lowest DFA item score was linked to scaling and polishing (mean: 1.59, score range: 1–5).

**Table 4:** Mean (Std. Err.) DFA scores for specific dental situations assessed by MDAS + FIS

Item	Weighted mean (SD)	Std. Err.	95% CI	Five-point scale (% weighted)				
				1	2	3	4	5
Visit tomorrow	1.71 (0.89)	0.01	1.68–1.73	52.58	28.56	15.25	2.22	1.29
Waiting room	1.85 (0.94)	0.01	1.83–1.87	45.61	30.35	18.69	4.23	1.12
Drill	2.79 (1.21)	0.01	2.76–2.82	16.49	24.75	34.00	12.75	12.02
Scale and polish	1.59 (0.91)	0.01	1.57–1.62	62.62	20.43	13.70	1.44	1.81
Injection	3.13 (1.32)	0.02	3.10–3.17	14.76	15.57	32.23	16.45	21.00
Total	11.07 (3.46)	0.04	10.99–11.15					

Unadjusted bivariate associations (survey-weighted linear regression) showed older students (11–12 years vs. 8–10) had 1.11 lower DFA scores (95% CI: -1.24 to -0.98), private vs. public school students had 0.98 lower scores (95% CI: -1.14 to -0.83), higher grades/birth order showed negative associations, and sex was non-significant. Adjusted multivariate model (F_(8,18)_ = 75.45, *p* < 0.001) confirmed these descriptive associations, as older students (β = -1.15, 95% CI: -1.41 to -0.89), private schools (β = -1.02, 95% CI: -1.15 to -090), and birth order 3+ (β = -0.73, 95% CI: -0.97 to -0.50) remained strongest predictors. Multivariate model assumptions were validated (Supplementary Appendix F) (Table 5).

**Table 5:** Survey-weighted linear regression of DFA score

Predictor		Bivariate β (95% CI)	Multivariate β (95% CI)
Age (vs. 8–10 years)	11–12	-1.11 (-1.24, -0.98)^**^	-1.15 (-1.41, -0.89)^**^
Sex (vs. boys)	Girls	0.03 (-0.10, 0.17)	-0.05 (-0.19, 0.09)
Grade levels (vs. grade 3)	Grade 4	-0.73 (-0.95, -0.50)^**^	-0.73 (-0.97, -0.50)^**^
	Grade 5	-1.63 (-1.89, -1.38)^**^	-0.98 (-1.28, -0.67)^**^
	Grade 6	-1.42 (-1.65, -1.19) ^**^	0.04 (-0.30, 0.38)
Birth order (vs. 1st )	2nd born	-0.23 (-0.43, -0.04)^*^	-0.25 (-0.43, -0.06)^*^
	3rd + born	-0.63 (-0.91, -0.35) ^**^	-0.73 (-1.04, -0.41) ^**^
School type (vs. public)	Private	-0.98 (-1.14, -083) ^**^	-1.02 (-1.15, -090) ^**^

^*^p < 0.05, ^**^ *p* < 0.001

R^2^ = 0.056, F(8,18) = 75.45, (*p* < 0.001), AIC=-4133. Model diagnostics confirmed validity with survey weights

Bivariate survey-weighted ordered logit (crude associations) showed statistically significant associations between sociodemographic and DFA levels (Table 6). Older children had lower odds of higher DFA category (11–12 vs. 8–10 years), with a crude odds ratio (COR) of 0.55 (95% CI: 0.52–0.60). Girls had higher odds of higher DFA levels than boys (COR = 1.12, 95% CI: 1.04–1.19). A clear decreasing trend in DFA was observed across grade level; compared with third graders (reference), students in grades 4, 5, and 6 had progressively lower odds of higher DFA (COR = 0.63, 95% CI: 0.56–0.70; COR = 0.38, 95% CI: 0.32–0.44; and COR = 0.43, 95% CI: 0.39–0.49, respectively). For birth order, third and later-born children had lower odds of higher DFA than first-born children (COR = 0.72, 95% CI: 0.63–0.83), whereas second-born children showed no significant difference. Children in private schools had lower odds of higher DFA than public school students (COR = 0.66, 95% CI: 0.60–0.72).

**Table 6:** Crude and adjusted odds ratio for the association between sociodemographic characteristics and DFA levels

Predictors		COR (95% CI)	GOR: DFA 0 vs. 1–2 (95% CI)	GOR: DFA 0–1 vs. 2 (95% CI)
Age	8–10 (ref.)	1.00 (ref.)	1.00 (ref.)	1.00 (ref.)
	11–12	0.55 (0.52, 0.60)^**^	0.41 (0.34, 0.49)^**^	0.62 (0.52, 0.75)^**^
Sex	Boys (ref.)	1.00 (ref.)	1.00 (ref.)	1.00 (ref.)
	Girls	1.12 (1.04, 1.19)^*^	1.98 (1.54, 2.55)^**^	1.04 (0.96, 1.13)
Grade	3 (ref.)	1.00 (ref.)	1.00 (ref.)	1.00 (ref.)
levels	4	0.63 (0.56, 0.70)^**^	0.89 (0.65, 1.23)	0.62 (0.54, 0.70)^**^
	5	0.38 (0.32, 0.44)^**^	1.55 (1.08, 2.24)^*^	0.45 (0.37, 0.56)^**^
	6	0.43 (0.39, 0.49)^*^	6.38 (3.91, 10.42)^**^	0.70 (0.55, 0.88)^*^
Birth	1 (ref.)	1.00 (ref.)	1.00 (ref.)	1.00 (ref.)
order	2	1.01 (0.90, 1.12)	0.45 (0.32, 0.63)^**^	1.07 (0.96, 1.20)
	3 or more	0.72 (0.63, 0.83)^**^	0.42 (0.29, 0.61)^**^	0.70 (0.60, 0.82)^**^
School	Public (ref.)	1.00 (ref.)	1.00 (ref.)	1.00 (ref.)
type	Private	0.66 (0.60, 0.72)^**^	0.66 (0.52, 0.85)^*^	0.66 (0.61, 0.72)^**^

^*^p < 0.05, ^**^ *p* < 0.001

*COR* Crude odds ratio (survey-weighted ordered logit), *GOR* Adjusted odds ratio (survey-weighted generalised ordered logit). Threshold-specific GORs are shown because the generalised ordered logit allows effects to differ across cumulative logits

Survey-weighted generalised ordered logit (*svy: gologit2*) multivariate analysis confirmed proportional odds violations via the global Wald test and provided threshold-specific adjusted associations (Supplementary Appendix G) [43]. Older students had lower odds of any DFA vs. no DFA (adjusted odds ratio of generalised ordered logit/GOR = 0.41, 95% CI: 0.34–0.49) and lower odds of high DFA vs. mild DFA (GOR = 0.62, 95% CI: 0.52–0.75). Girls (vs. boys) had higher odds of any DFA vs. no DFA (GOR = 1.98, 95% CI: 1.54–2.55) but similar odds of high vs. mild DFA (GOR = 1.04). Among grades (vs. grade 3), fourth-graders had similar odds across thresholds. The fifth graders had higher odds of any DFA vs. no DFA (GOR = 1.55, 95% CI: 1.08–2.24) but lower odds of high vs. mild DFA (GOR = 0.45, 95% CI: 0.37–0.56). Sixth-graders had dramatically higher odds of any DFA vs. no DFA (GOR = 6.38, 95% CI: 3.91–10.42) but lower odds of high DFA vs. mild DFA (GOR: 0.70, 95% CI: 0.55–0.88). Second-born and later-born (3+) children (vs. first-born) had lower odds of any DFA vs. no DFA (GOR = 0.45 and 0.42, respectively). Private school students (vs. publics) had lower odds across both thresholds (GOR = 0.66).

Across all models, private school attendance and older age consistently showed the strongest inverse adjusted associations with DFA levels. Higher birth order categories demonstrated progressively lower DFA. Threshold-specific grade associations in *gologit2* refined but did not contradict these patterns. Sensitivity analyses further confirmed robustness (Supplementary Appendix H). For instance, tertile-based cut-offs in *gologit2* yielded consistent sociodemographic associations compared to median-based cut-offs. Household expenditure adjustment preserved all main findings across both linear and *gologit2* models, with linear regression retaining significance for all key variables, and *gologit2* maintaining directional consistency despite reductions in magnitude and some changes in *p*-values.

The analysis presented here focuses on the descriptive association between total DFA score and sociodemographic variables. A sensitivity analysis adjusting for household expenditure shows that the association remains robust but slightly attenuated (Supplementary Appendix H), indicating that school type partly reflects underlying socioeconomic context but cannot fully replace adjustment for family-level confounders.

**Fig. 1:**
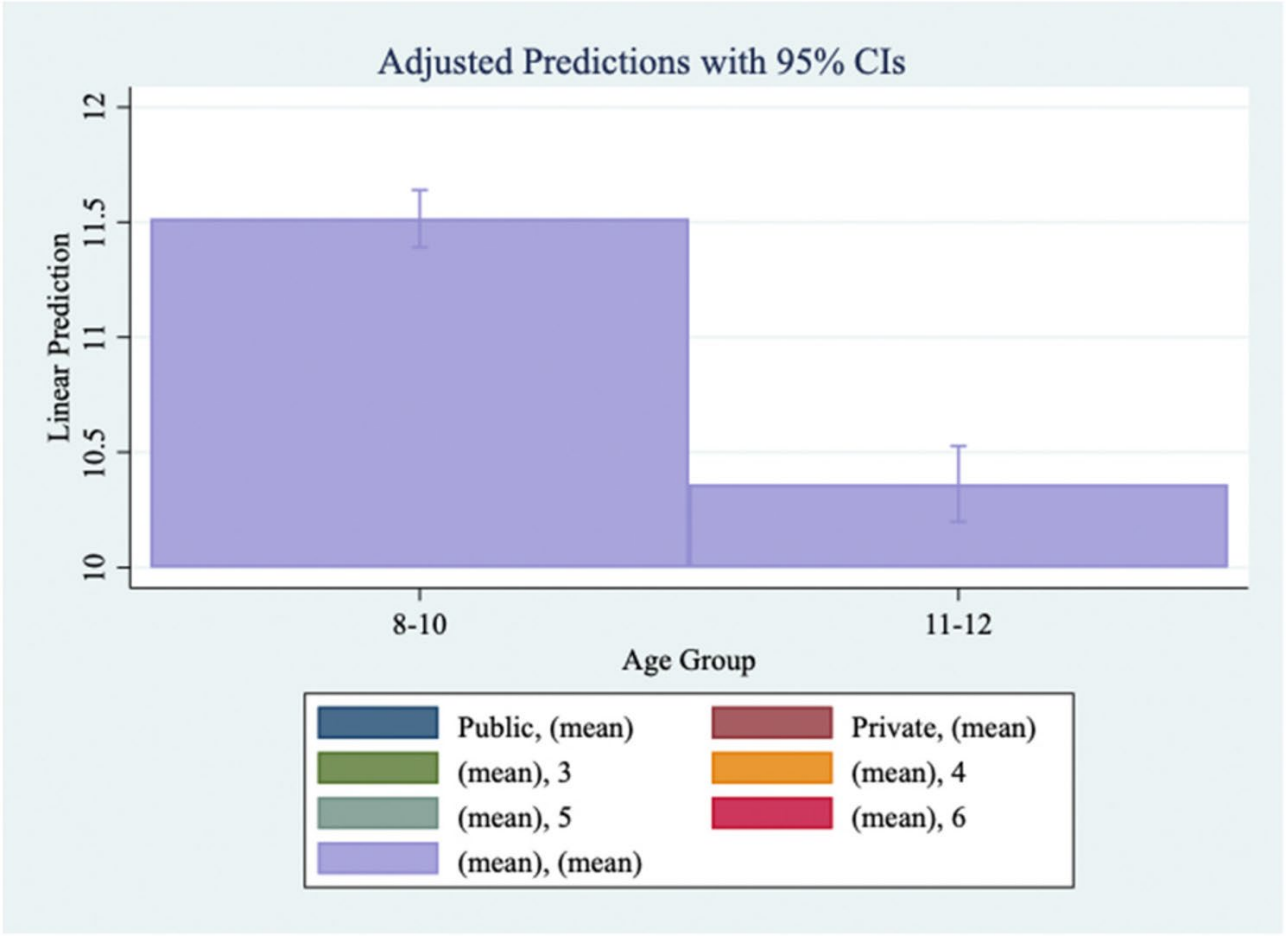
Predicted DFA score by age group

**Fig. 2:**
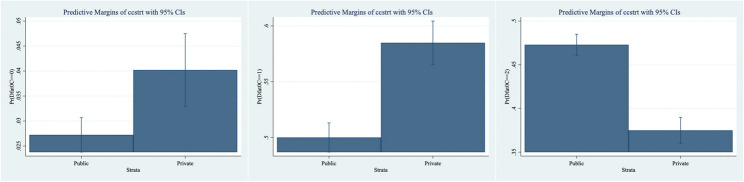
Predicted probabilities of no DFA, mild DFA, and high DFA across school types

The survey-weighted linear regression model (margins with age, school type, grade; covariates at means) yielded predicted DFA scores that were consistently lower among older students (11–12 years) than younger students across all grade levels and school types (Fig. 1). Additionally, private school students exhibited higher predicted probabilities (using survey-weighted generalised ordered logit) of both no DFA and mild DFA, alongside lower probabilities of high DFA, compared to public school students across *gologit2* thresholds (Fig. 2), consistent with linear regression findings.

## Discussion

This study presents baseline DFA data among Depok City schoolchildren aged 8–12 years. The total DFA score was analysed as both a continuous and a categorical variable. Consistent associations with older age, private schooling, and higher birth order were identified across models, with school type demonstrating the strongest and most robust inverse association across all model specifications.

Most children showed some DFA (mild 53.2%, high 43.6%), suggesting its relatively high prevalence in this population. The median cut-off categorisation yielded a higher prevalence of high-DFA (43.6%) than the internationally reported common MDAS thresholds (3–36%) [44–47]. This reflects our sample’s DFA distribution, as confirmed by tertile sensitivity analysis showing stable sociodemographic patterns across cut-offs.

This median cut-off categorisation was chosen because there is currently no established MDAS cut-off for children in Indonesia and international studies use varying cut-off values, categorisations, and labelling [44–46, 48]. To illustrate the variability in MDAS-based categorisations, studies have used different cut-offs, categorisations, and labels. One study defined MDAS scores below 10 as “no to low dental anxiety”, between 11 and 18 as “moderate anxiety”, and 19 or above as “high anxiety” [44]. Another dichotomised MDAS at 19, treating scores below 19 as “no anxiety” and 19 or higher as “dental anxiety” [48]. A third used a cut-off of 13 to distinguish “anxious” from not “anxious” [45]. A fourth classified scores of 10 or less as “normal”, 11–18 as “moderate anxiety”, and 19 or above as “extreme anxiety” [46]. These examples highlight how MDAS cut-offs, DFA-level terminology, and categorisation vary substantially across studies. While direct comparisons are limited by methodological heterogeneity across studies (assessment tools, cut-off thresholds, sociodemographic settings) [6, 11, 15, 16, 20, 25, 35, 44–52], these findings highlight DFA’s high prevalence among Indonesian schoolchildren, potentially contributing to low dental attendance rates.

Dental injections and dental drilling were primary DFA triggers, consistent with previous studies [6, 25, 47, 53]. These fears may stem from fear conditioning or pain memories associated with the needle or the dental drill [6, 11, 54–56]. Therefore, a positive first dental experience is crucial for children, particularly at an early age. Dentists should prioritise pain minimisation, for example, by using less painful needles or adopting atraumatic restorative treatment [57, 58].

The level of DFA was higher among younger children compared to their older counterparts. This finding aligns with several studies [17, 20, 25, 26, 51], although some have reported the opposite or no association [6, 16, 19, 24, 50]. From a psychological perspective, the higher DFA levels observed in younger children may be attributed to their intellectual development stage and limited understanding of dental settings [54]. Similarly, lower school grade was associated with higher DFA, suggesting that the transition to formal schooling may heighten apprehension toward unfamiliar dental scenarios [59]. This period coincides with the development of social-emotional skills, during which children may be influenced by negative peer information [60].

The present study also identified significant associations between DFA, birth order, and school type. Sex showed mixed findings, as girls had slightly higher odds of exhibiting DFA in ordered logit and markedly higher odds in *gologit2*, though linear regression found no significant difference. Previous literature showed similar inconsistency, with some studies demonstrating girls were more likely to experience DFA than boys [20, 21, 59], while others reported contradictory or no evidence [5, 6, 23].

Later-born children were less likely to demonstrate high levels of DFA, consistent with previous research [19, 27], possibly reflecting observational learning from older siblings. Prior studies reported that sibling presence was associated with increased DFA [19, 61, 62], whereas others have found no such evidence [23, 26], suggesting that cultural factors may moderate these associations. In the present study, siblings appeared to function not only as behavioural models but also as potential protective factors by providing emotional support or bravery reinforcement. This interpretation, however, warrants further investigation. Conversely, in certain cultural settings, sibling interactions may amplify DFA through teasing or intimidation.

Children enrolled in public schools were more likely to display higher levels of DFA, suggesting socioeconomic disparities are associated with children’s DFA, consistent with previous studies [23, 24, 50, 59]. In Depok City, public schools have lower fees while private schools charge higher costs with better resources. School type serves as a proxy for family socioeconomic status (SES) in this study, as family SES (e.g., household expenditure and parental education) were excluded in the main analysis to focus on children’s individual attributes. Sensitivity analyses that included household expenditure in the models showed that the association between school type and DFA remained robust.

Studies showed that untreated dental caries were more common among children from public schools or lower socioeconomic groups [4, 9], increasing the likelihood of painful treatment due to delayed care. In contrast, children from higher socioeconomic groups typically receive regular dental care, potentially contributing to lower DFA among Depok City private-school students. One study found school type unrelated to dental fear prevalence, although family income was significant [17], suggesting that direct SES indicators may capture certain aspects of the underlying socioeconomic gradient more clearly than school type alone.

The current analysis reports the descriptive association between school type and DFA at the context level, as well as between DFA and age, sex, birth order, and grade. These results highlight key observed relationships. Adjustment for family confounders will be addressed in a planned secondary analysis. Further research should also examine additional sociodemographic dimensions, including family structure and urban versus rural context.

Key strategies to highlight include the quality of communication among young patients, parents, and the dentist. Evidence shows that empathetic and supportive communication, as well as early positive introduction to dental settings can significantly reduce children’s DFA [6, 16, 17, 20]. Establishing this constructive interaction is therefore fundamental in creating a more reassuring dental experience.

Early introduction to dental care, before the onset of oral health issues, is recommended as part of promotive and preventive strategies to develop a positive dental experience and long-term oral health. Early interventions, such as a school-based program, could mitigate DFA in this age group, with designs tailored for easy comprehension by children at varying stages of cognitive development [19, 20, 50, 59]. The main challenge lies in developing broadly applicable, cost-effective strategies involving schools, parents, communities, and dental practitioners. These must adapt to local sociocultural contexts, while also engaging and appealing to the targeted population.

Strengths of the present study include a representative sample size, which enabled reliable statistical analysis, and the use of the validated Indonesian MDAS, supplemented by FIS to minimise response bias. Training for supervisors and enumerators ensured data collection consistency and reduced interviewer bias. Pilot testing confirmed instrument reliability, reducing instrument-related bias, while conducting interviews in familiar school settings promoted relaxed responses and more genuine answers. A complete-case analysis was appropriate given low, concentrated missingness (< 3% of dataset cells), high per-variable completeness (> 94%), and no missing primary outcome (DFA) or covariates, which minimised bias while retaining 92.74% of dyads [41]. Weights matched grade 3–6 structure from *Dapodikdasmen*, enhancing generalisability to Depok City’s schoolchildren population. Cell-based nonresponse weighting was infeasible after exclusions, but the calibrated design and low missingness support robust estimates.

This study’s limitations include its cross-sectional design, which limits findings to only descriptive associations. The findings apply only to elementary schools in Depok City, given its urbanising peri-urban setting and distinct sociodemographic profile. The analysis excludes other potential confounders, such as children’s prior dental visit experience and parental DFA [19, 25]. Enumerator-administered questionnaires may still introduce interviewer bias despite quality control measures.

Based on the findings of the present study and the existing literature, several important issues warrant further consideration in the field of DFA. First, there is a need for universal standardisation of terminology related to the phenomenon to facilitate clearer communication and interpretation across studies. Second, the use of diverse psychometric instruments and varying cut-off thresholds hinders the comparability of DFA levels between different populations. Third, there is a need for a clearly defined spectrum to classify the severity of DFA globally, ideally supported by physiologically objective measurement tools to complement self-reported assessments.

## Conclusions

This study confirms a significant prevalence of mild and high DFA among Depok City elementary schoolchildren aged 8–12 years, with lower DFA among older children (age 11–12 years), higher grade levels, later birth order, and private school attendance. Dental injections and dental drilling were major triggers. These findings establish baseline data and highlight the need for locally tailored early interventions, particularly for younger children and public-school students, through empathetic dental communication and early positive dental experiences to improve long-term oral health among schoolchildren.

## Supplementary Information

Supplementary Material 1.

## Data Availability

The data that support the findings of this study are available on request from the corresponding author, WF. The data are not publicly available due to their containing information that could compromise the privacy of research participants under Indonesia’s Law No. 27 of 2022 on Personal Data Protection (PDP).

## Abbreviations

DFA: Dental Fear and Anxiety
ES: Elementary School(s)
MDAS + FIS: Indonesian Modified Dental Anxiety Scale integrated with the Facial Image Scale
Riskesdas: Riset Kesehatan Dasar (The Indonesian National Basic Health Research)
Susenas: Survei Sosial Ekonomi Nasional (The Indonesian National Socioeconomic Survey)
I-CVI: Item-level Content Validity Index
PPS: Probability Proportional to Size
FPC: Finite Population Correction
