# Supplementary material for: Dental fear and anxiety and its sociodemographic associations among public and private schoolchildren aged 8 to 12 years in Depok, Indonesia: a cross-sectional study
Source: BMC Oral Health. 2026 May 11;26:1207. doi: 10.1186/s12903-026-08481-6 (PMC13343720; doi:10.1186/s12903-026-08481-6)
Supplement: Supplementary file 1 — Supplementary Material 1. [file 12903_2026_8481_MOESM1_ESM.docx]

**Supplementary Appendix A: Conceptual Framework and Confounding Factors**

Figure SA1 presents a simple conceptual framework for the association between school type (public vs private) and dental fear and anxiety (DFA) in children aged 8–12 years in Depok. This framework considers several sociodemographic factors, including family expenditure, parental education, parental age, household composition, and other family sociodemographic attributes, as potential confounders of the school‑type–DFA association.

Family‑level sociodemographic variables, such as financial capacity, parental education, and access to dental care, are expected to influence the decision to enrol a child in a public or private elementary school and the child’s DFA. Simultaneously, school type may also operate as a contextual proxy for socioeconomic status, given that private‑school enrolment in Depok City is largely driven by household affordability and parental educational priorities.

This study focuses on the descriptive association between school type and DFA, adjusted for age, sex, birth order, and grade levels. A full adjustment for all family sociodemographic factors (such as family expenditure, parental education, parental age, household composition, and other sociodemographic attributes) was not possible at this stage due to the cross‑sectional design and the planned separation of analyses across papers. Thus, this conceptual framework supports an interpretation of the school‑type association as a context‑level marker of underlying socioeconomic context, rather than a fully confounder‑adjusted association.

Figure SA1. Simple Conceptual Framework


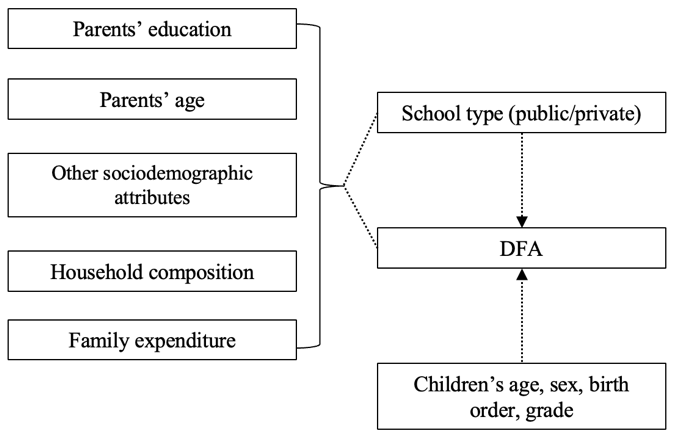


In this framework, family (household) expenditure, parents’ education, parents’ age, household composition, and other family sociodemographic attributes are considered potential confounders of the association between school type and DFA. School type reflects the socioeconomic context in which children are embedded in education and society. Age, sex, birth order, and grade level are included as adjustments in the main analysis to account for individual‑level differences.

Despite measuring these family‑level variables, the present analysis intentionally focuses on the descriptive association between DFA and the covariates (age, sex, birth order, grade level, and school type). The planned secondary analysis will explore the independent contribution of family‑level socioeconomic variables to DFA to provide a more nuanced understanding of the confounding factors.

**Supplementary Appendix B: Instrument Description**

Dental fear and anxiety (DFA) were measured using the MDAS+FIS, an integrated adaptation of the validated Indonesian Modified Dental Anxiety Scale (MDAS) and the Facial Image Scale (FIS), modified for children aged 8–12 years.

The MDAS+FIS consists of five items that refer to common dental situations: (1) anticipating a dental visit tomorrow; (2) waiting in the dental clinic; (3) having a tooth drilled; (4) having teeth scaled or polished; and (5) receiving a local anaesthetic injection. Each item is rated on a five‑point Likert scale from 1 = not anxious at all to 5 = extremely anxious, with smiley‑face illustrations (FIS) attached to each response option: 1 = biggest smile (no anxiety); 2 = smaller smile, 3 = neutral expression, 4 = small frown, and 5 = extreme frown (extreme anxiety).

The total DFA score is the sum of the five items, with a theoretical range of 5–25. A higher total score indicates greater DFA.

The wording of the Indonesian MDAS+FIS items was based on the existing Indonesian MDAS version published by the University of St Andrews, with slight revisions to facilitate child comprehension while retaining the meaning. The full MDAS+FIS questionnaire block (Block IV of the child questionnaire) in both the Bahasa Indonesia and English versions is reproduced in this appendix (**Figure SA2** and **Figure SA3**).

Figure SA2. Block IV of the child questionnaire: MDAS+FIS in Bahasa Indonesia


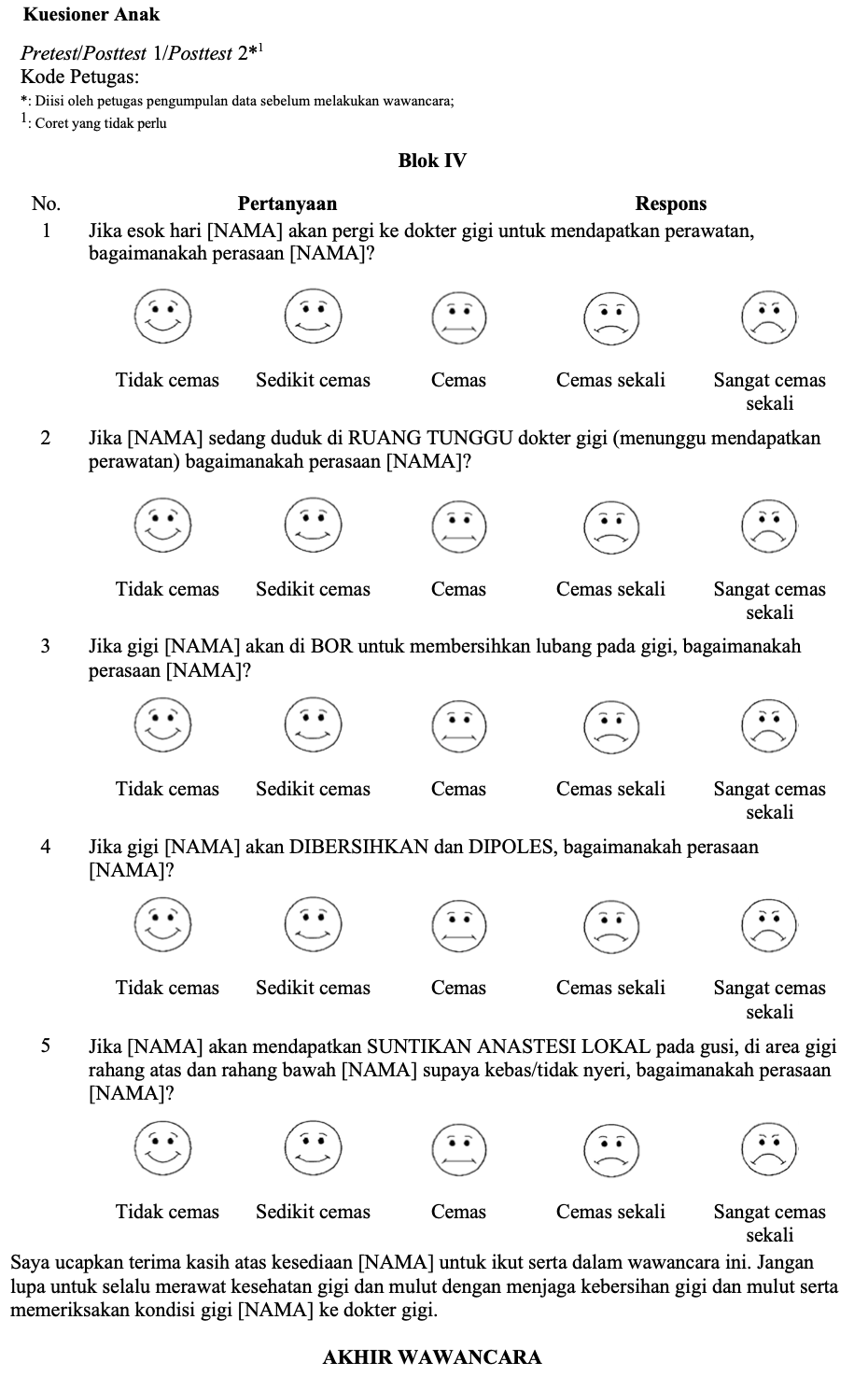


Figure SA3. Block IV of the child questionnaire: MDAS+FIS in English


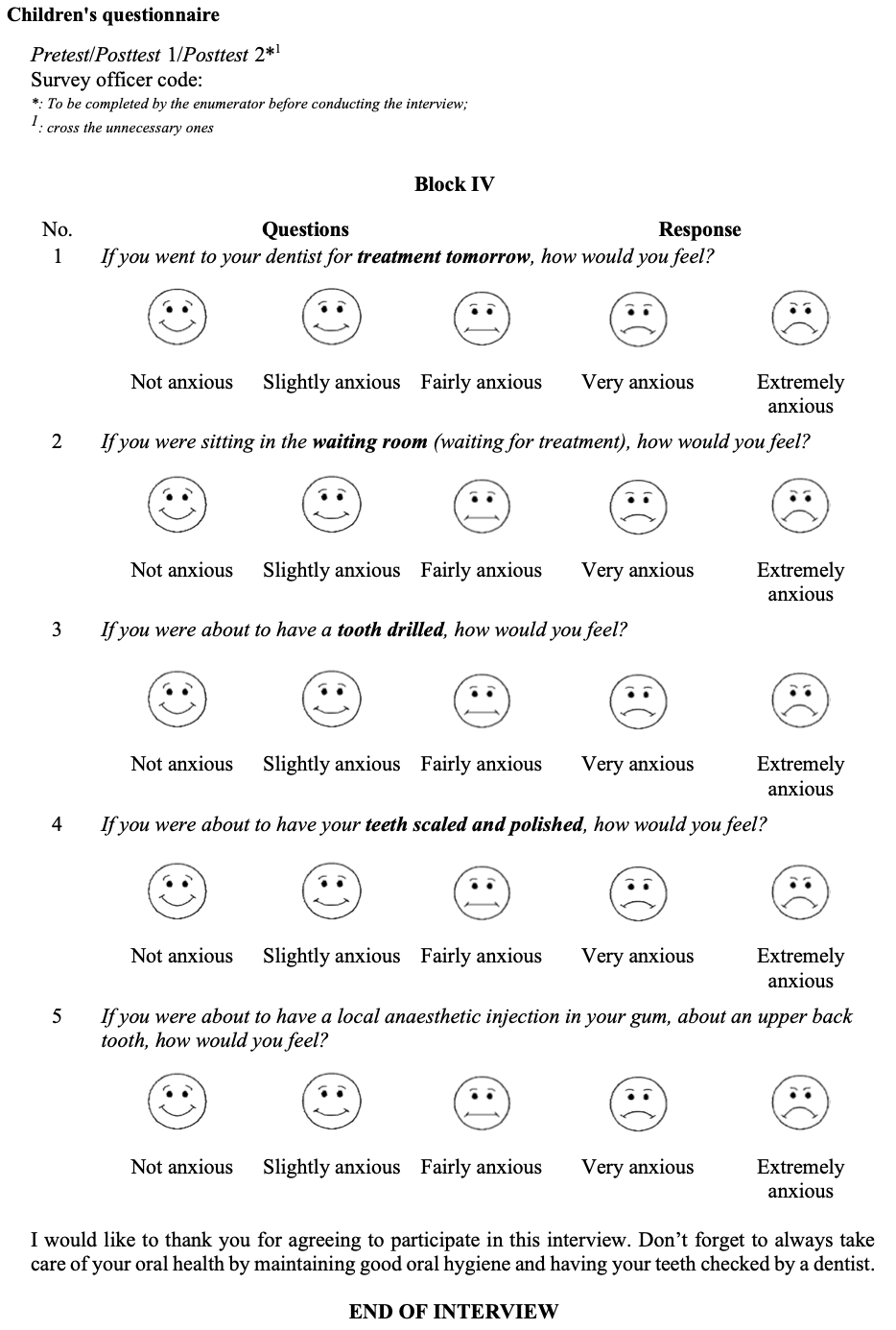


**Supplementary Appendix C: Instrument Validity and Reliability**

Instrument validity and reliability were assessed before full‑scale fieldwork to minimise instrument‑related bias and ensure that the adapted tools yielded consistent and meaningful measurements. The procedures followed a multi‑step approach, including content validity assessment by subject‑matter experts and test–retest reliability evaluation using a purposively selected pilot sample.

Three community dentistry experts assessed the content validity of the child and parent questionnaires. Each expert independently rated the relevance and appropriateness of each item using a 3‑point ordinal scale (e.g., disagree, undecided, and agree). The rating categories were interpreted as follows: “disagree” indicated that the item was not relevant or appropriate, “undecided” implied uncertainty, and “agree” denoted that the item was clearly relevant and appropriate. The item-level Content Validity Index (I‑CVI) for the 3-point rating scale was computed as the proportion of experts who rated an item as “agree”. All items in the child and parent questionnaires, including sociodemographic items and other domains of interest, yielded an I-CVI of 1.00. The three experts agreed that each item was relevant and appropriate, indicating that the adapted instruments had strong content. The DFA-specific items (Q1–Q5) appear only in the child questionnaire (Block IV), whereas no DFA-specific items are included in the parent questionnaire.

Internal reliability of the five DFA-related items (Q1-Q5) in the child questionnaire at Time 1 was assessed using Cronbach’s alpha and ordinal alpha (polychoric alpha), with the latter being preferred for Likert-type ordinal data as it accounts for the ordered categorical nature of the response. The ordinal alpha was 0.775 (*n* = 31), indicating good consistency (≥ 0.7 is acceptable; ≥ 0.8 is preferable), whereas the standard Cronbach’s alpha was 0.704.

A pilot sample was drawn from two fourth-grade classes (children aged 9–10 years) at one unselected elementary school in Depok City. These classes were purposively selected to represent the main survey’s age group while remaining geographically similar to the overall study sample. Test-retest reliability was evaluated in this sample. A total of 64 children and their parents were initially selected. However, not all participants completed both questionnaire administrations due to logistical and scheduling constraints. The test-retest interval for the child interview was approximately 4 h on the same day, whereas that for the parent interview was approximately 6–8 h on the same day. The aim was to minimise the likelihood of genuine change in children’s DFA over time while allowing sufficient time to reduce immediate memory‑recall effects. This short‑term test–retest design enabled the assessment of individual responses’ temporal stability under comparable conditions.

Reliability was assessed using different metrics based on item type. For categorical items (e.g., yes/no or unordered categories), the agreement between Time 1 and Time 2 was quantified using Cohen’s kappa (*κ*), which adjusts for chance agreement. For ordinal DFA‑related items in the child questionnaire (Likert‑type rating scales), the strength of the latent association between responses at the two time points was estimated using polychoric correlation (*ρ*), assuming an underlying continuous latent variable.

The results were as follows:

1. Children’s questionnaire, Blocks I–III (core sociodemographic and other domains of interests; *n* = 39 children who completed both administrations):

- Cohen’s kappa ranged from approximately κ ≈ 0.60 to κ = 1.00 across repeated categorical items.
- All κ values were statistically significant (*p* < 0.001), indicating moderate to perfect agreement and good temporal stability.

1. Parent questionnaire (sociodemographic, dental‑knowledge, and dental‑practice items; *n* = 32 parents who completed both administrations; no DFA‑related items):

- Cohen’s kappa ranged from approximately κ ≈ 0.79 to κ = 1.00
- All values were statistically significant (*p*<0.001), indicating a predominantly perfect agreement and high temporal stability of parental responses.

1. Children’s questionnaire, Block IV/DFA‑related Likert items (DFA‑scale items using Likert‑type responses; *n* = 31 children with complete test–retest data):

- The polychoric correlations ranged from approximately *ρ* ≈ 0.83–1.00, indicating strong to perfect latent consistency over time.
- The model fit for these DFA‑related items, assessed using the Pearson G² goodness‑of‑fit statistic, showed *p* ≥ 0.389, supporting a good fit of the underlying factor structure and reinforcing the construct reliability of the DFA‑related scale.

These results support the temporal stability and construct reliability of the MDAS+FIS instrument.

**Supplementary Appendix D: Survey Design and Weighting**

The study used a stratified, three‑stage cluster design with classes serving as the primary sampling units (PSUs) and school type (public vs private) as the stratifying factor. Two subdistricts were randomly selected from the 11 subdistricts in Depok City, each with an equal probability of being selected. Five elementary schools were randomly selected from 42 schools in the selected subdistricts with equal probability in stage 2. A total of 27 classes, each with equal probability, were randomly selected from 66 classes in the selected schools

**Finite population correction**

Finite population correction (FPC) was applied at the subdistrict stage (*fpc1*) using *Dapodikdasmen* pupil counts for the total elementary-school-age population and the number of pupils in the two selected subdistricts. *Dapodikdasmen* (*Data Pokok Pendidikan Dasar dan Menengah*/Primary and Secondary Education Core Data) is the Indonesian Ministry of Education’s national database. The database reports the total number of classes and pupil counts per school.

The FPC (*fpc1*) term was computed as:

$$\sqrt{{(159,023-14,930)}/{(159,023-1)}}$$

**Weight calculation**

The sampling used equal probabilities at each stage, and the weights were constructed using the probability-proportional-to-size (PPS) approach based on pupil counts at the subdistrict, school, and class levels. Sampling weights were computed as:

$${=1}/{({pr}_{1}*{pr}_{2}*{pr}_{3}})$$

where pr_1,_ pr_2,_ and pr_3_ are the subdistrict, school, and class selection probabilities, respectively. The final weights were implemented in Stata using the *svyset* command with *fpc1* (FPC at the subdistrict stage).

**Weight calibration**

The final weights were calibrated against the total population of Depok City’s pupils in grades 3–6, which corresponds approximately to 2/3 of the total elementary‑school‑age population (grades 1–6) in the city. This calibration margin was chosen because the study included only children aged 8–12 years, who were predominantly enrolled in grades 3–6 in the Depok education system. Calibration was implemented as a scalar adjustment: the base design weights were scaled so that the sample weighted sum matched the target population size for grades 3–6. This ensures that the weighted estimates are representative of the broader population of children in those grades while accounting for the subdistrict, grade, and sex structure.

**Supplementary Appendix E: DFA Score Distribution**

The histogram shows the distribution of DFA scores among children aged 8–12 years in Depok (**Figure SA4**). The median score is 11, with 25th and 75th percentiles of 9 and 13, respectively. The 90th percentile is 16, and the 95th percentile is 17, indicating that most children have DFA scores in the mild range. The 1st percentile is 5, and the 99th percentile is 20 (**Table SA1**).

Figure SA4. Distribution of the total DFA score (MDAS)

**
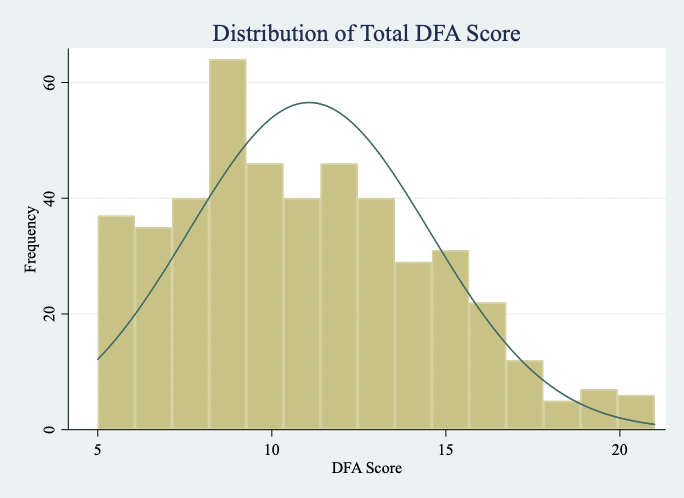
**

Table SA1. Percentiles of the total DFA score

| **Percentile** | **DFA Score** |
| --- | --- |
| 1^st^ | 5 |
| 2^nd^ | 6 |
| 10^th^ | 7 |
| 25^th^ | 9 |
| 50^th^ | 11 |
| 75^th^ | 13 |
| 90^th^ | 16 |
| 95^th^ | 17 |
| 99^th^ | 20 |
| N = 460 (total sample) | |
| Range = 5–21 | |

**Supplementary Appendix F: Multivariate Linear Model Assumptions Tests**

The multivariate linear regression model diagnostics confirm the validity of key assumptions. The residual histogram demonstrates approximate normality, with a symmetric, bell-shaped distribution centred near zero, supporting reliable inference. The Q-Q plot shows residuals closely aligned with the diagonal reference line and minimal deviations in the tail, further validating the normality and the absence of strong outliers (**Figure SA5**). The residuals vs fitted values plot shows random scatter around the zero line across the full range of fitted values, with no funnel pattern, curvature, or clustering. This confirms the homoscedasticity assumption, as the variance appears stable across predicted DFA scores.

Figure SA5. Linear regression model diagnostics: Residual histogram, Q-Q plot, and residual vs fitted values plot


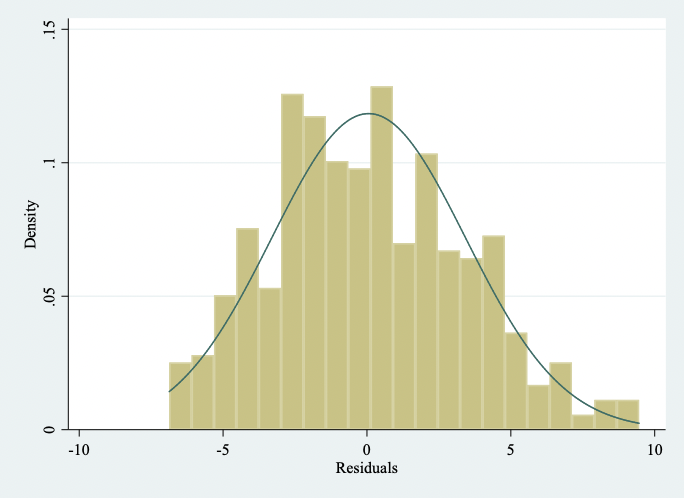

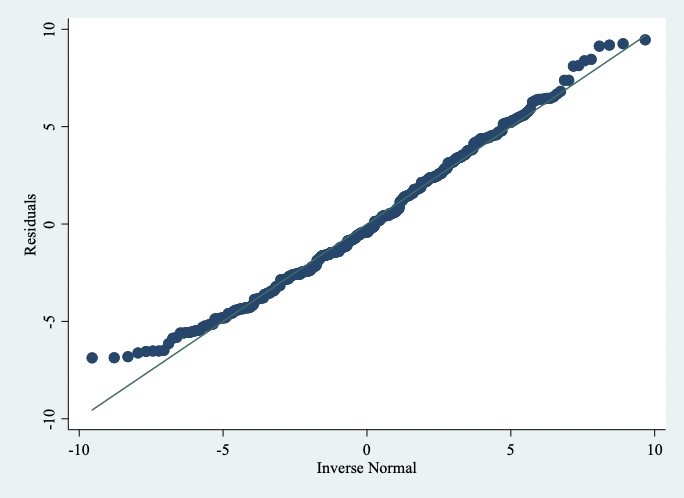

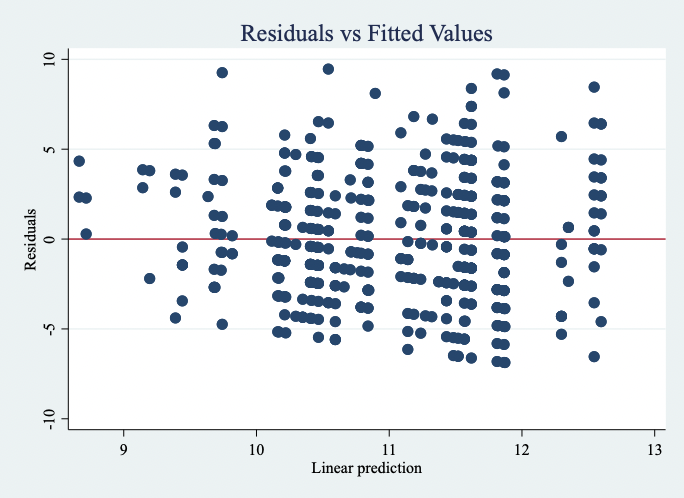


**Supplementary Appendix G: Proportional Odds Assumptions Test**

In the initial multivariate survey-weighted ordered logistic regression, younger students (8–10 years) had higher odds of a higher DFA category than the older students (11–12 years), with an adjusted odds ratio (AOR) of 0.60 (95% CI: 0.51–0.71). Girls had slightly higher odds than boys (AOR = 1.08, 95% CI: 1.00–1.17). Among grade levels, third graders (reference) had the highest odds, while the fifth graders had the lowest adjusted odds (AOR = 0.49, 95% CI: 0.41–0.59). Later-born students (birth order 3+) had reduced odds compared to the first-born children (AOR = 0.68, 95% CI: 0.59–0.78). Private school students had markedly lower odds of higher DFA versus public school students (AOR = 0.66, 95% CI: 0.61–0.71). Overall, the model was highly significant with F_(8,18)_ = 81.42 and *p* < 0.001, confirming strong covariate associations with DFA levels in this survey population. This model, however, was excluded from the main manuscript as it violated the proportional odds assumption test (**Table SA2**)

Table SA2. Survey-weighted ordered logistic regression

| **Predictors** | | **COR (95% CI)** | **AOR (95% CI)** |
| --- | --- | --- | --- |
| Age | 8-10 (ref.) | 1.00 (ref.) | 1.00 (ref.) |
|  | 11-12 | 0.55 (0.52, 0.60)^**^ | 0.60 (0.51, 0.71)^**^ |
| Sex | Boys (ref.) | 1.00 (ref.) | 1.00 (ref.) |
|  | Girls | 1.12 (1.04, 1.19)^*^ | 1.08 (1.00, 1.17)^*^ |
| Grade | 3 (ref.) | 1.00 (ref.) | 1.00 (ref.) |
| levels | 4 | 0.63 (0.56, 0.70)^**^ | 0.62 (0.56, 0,70)^**^ |
|  | 5 | 0.38 (0.32, 0.44)^**^ | 0.49 (0.41, 0.59)^**^ |
|  | 6 | 0.43 (0.39, 0.49)^*^ | 0.79 (0.64, 0.98)^*^ |
| Birth | 1 (ref.) | 1.00 (ref.) | 1.00 (ref.) |
| order | 2 | 1.01 (0.90, 1.12) | 1.01 (0.91, 1.13) |
|  | 3 or more | 0.72 (0.63, 0.83)^**^ | 0.68 (0.59, 0.78)^**^ |
| School | Public (ref.) | 1.00 (ref.) | 1.00 (ref.) |
| type | Private | 0.66 (0.60, 0.72)^**^ | 0.66 (0.61, 0.71)^**^ |
| ^*^*p*<0.05, ^**^ *p*<0.001.  COR = crude odds ratio; AOR = adjusted odds ratio from survey-weighted ordered logistic regression; GOR = generalised odds ratio from survey-weighted generalised ordered logit. Threshold-specific GORs are shown because generalised ordered logit allows effects to differ across cumulative logits. | | | |

The proportional odds assumption was tested by comparing a constrained proportional-odds model (parallel-lines constraints across thresholds) with an unconstrained generalised ordered logit model using s Wald test (**Table SA3**). The null hypothesis was that the predictor coefficients were equal across the two models, implying that the proportional-odds assumption held. The global Wald test yielded a statistically significant result (F_(8,18)_ = 15.10, *p* < 0.05), indicating that the assumption was violated overall. Wald tests for individual predictors also showed significant differences between the constrained and unconstrained models (all *p* <0.005), providing evidence of threshold-specific associations and supporting the use of the generalised ordered logit model as the final multivariate model.

Table SA3 Proportional Odds Tests (Adjusted Wald Tests)

| **Predictors** | | **Wald F** | **p-value** | **Proportional Odds** |
| --- | --- | --- | --- | --- |
| Age | 11­–12 vs 8–10 | 11.15 | <0.005 | Assumption violated |
| Sex | Girls vs Boys | 22.09 | <0.001 | Assumption violated |
| Grade levels | 4 vs 3 | 20.07 | <0.001 | Assumption violated |
|  | 5 vs 3 | 24.24 | <0.001 | Assumption violated |
|  | 6 vs 3 | 24.34 | <0.001 | Assumption violated |
| Birth order | 2^nd^ vs 1^st^ | 21.33 | <0.001 | Assumption violated |
|  | 3+ vs 1^st^ | 17.94 | <0.001 | Assumption violated |
| School type | Private vs Public | 15.06 | <0.001 | Assumption violated |

**Supplementary Appendix H: Sensitivity Analyses**

Sensitivity analyses confirm the robustness of the main findings across alternative specifications. Table SA4 demonstrates that sociodemographic associations with DFA remain directionally consistent and statistically significant when using tertile-based cut-offs rather than median cut-offs in the generalised ordered logit model, though the magnitudes are slightly attenuated at the high DFA threshold. Table SA5 shows that adjusting the linear regression for monthly household expenditure slightly reduces school type coefficients while preserving significance across all key predictors, indicating school type captures both contextual and residual socioeconomic associations. Table SA6 confirms that household expenditure adjustment in the generalised ordered logit model maintains directional consistency across both thresholds despite some attenuation, particularly for private school associations, supporting the main model’s validity while acknowledging socioeconomic confounding pathways.

Table SA4 Sensitivity analysis – Selected coefficients by DFA cut-off (generalised ordered logit model, second cut-off point shown)

| **Sociodemographic  characteristics** | | **Main model (No DFA, Mild DFA, High DFA)** | **Tertile cut-off (Low DFA, Mild DFA, High DFA)** |
| --- | --- | --- | --- |
| Age | 8-10 (ref.) |  |  |
|  | 11-12 | -0.898^***^ | -0.476^***^ |
| Sex | Boys (ref.) |  |  |
|  | Girls | 0.685^***^ | 0.071 |
| Grade | 3 (ref.) |  |  |
| levels | 4 | -0.114 | 0.172^***^ |
|  | 5 | 0.439^*^ | 0.419^***^ |
|  | 6 | 1.853^***^ | 1.118^***^ |
| Birth | 1 (ref.) |  |  |
| order | 2 | -0.801^***^ | -0.199^**^ |
|  | 3 or more | -0.878^***^ | -0.359^**^ |
| School | Public ref.) |  |  |
| type | Private | -0.410^**^ | -0.564^***^ |
| ^*^*p*<0.05, ^**^ *p*<0.01, ^***^*p*<0.001 | | | |

Table SA5. Linear regression coefficients: Main model vs household expenditure sensitivity analysis

| **Variable** | **Linear model** | **Linear sensitivity model** |
| --- | --- | --- |
| **Age** |  |  |
| 11–12 | -1.153^***^ | -1.105^***^ |
| **Sex** |  |  |
| Girls | -0.052 | -0.037 |
| **Grade** |  |  |
| 4 | -0.731^***^ | -0.750^***^ |
| 5 | -0.977^***^ | -0.959^***^ |
| 6 | 0.040 | -0.044 |
| **Birth order** |  |  |
| 2 | -0.246 | -0.194^*^ |
| 3+ | -0.725^***^ | -0.705^***^ |
| **School type** |  |  |
| Private | -1.025^***^ | -0.903^***^ |
| **Household expenditure** |  |  |
| Rp. 1–2.5 million | na | 0.491^**^ |
| Rp. 2.5–5 million | na | 0.370^**^ |
| Rp. 5–10 million | na | 0.404^*^ |
| Rp. 10 million+ | na | -0.440 |
| **_cons** | 12.597^***^ | 12.180^***^ |
| ^*^ p<0.05; ^**^ p<0.01; ^***^ p<0.001; na: not applicable | | |

Table SA6. Gologit2 coefficients by threshold: Main model vs household expenditure sensitivity analysis

| **Variable** | **Gologit model (Threshold 1)** | **Gologit sensitivity model (Threshold 1)** | **Gologit model (Threshold 2)** | **Gologit sensitivity model (Threshold 2)** |
| --- | --- | --- | --- | --- |
| **Age** |  |  |  |  |
| 11–12 | -0.898^***^ | -1.206 | -0.475^***^ | -0.440 |
| **Sex** |  |  |  |  |
| Girls | 0.685^***^ | 0.771 | 0.040 | 0.042 |
| **Grade** |  |  |  |  |
| 4 | -0.114 | 0.055 | -0.486^***^ | -0.518 |
| 5 | 0.439^*^ | 0.725 | -0.788^***^ | -0.778 |
| 6 | 1.853^***^ | 2.444 | -0.362^**^ | -0.447 |
| **Birth order** |  |  |  |  |
| 2 | -0.801^***^ | -0.913 | 0.068 | 0.122 |
| 3+ | -0.878^***^ | -0.905 | -0.353^***^ | -0.336 |
| **School type** |  |  |  |  |
| Private | -0.410^**^ | -0.798 | -0.418^***^ | -0.291 |
| **Household expenditure** |  |  |  |  |
| Rp. 1–2.5 million | na | -18.019 | na | 0.429 |
| Rp. 2.5–5 million | na | -17.292 | na | 0.289 |
| Rp. 5–10 million | na | -17.101 | na | 0.291 |
| Rp. 10 million+ | na | -16.786 | na | -0.612 |
| **_cons** | 3.792^***^ | 21.224 | 0.511^***^ | 0.191 |
| ^*^ p<0.05; ^**^ p<0.01; ^***^ p<0.001; na: not applicable | | | | |
